# Extended Reality for Neuraxial Procedures: A Scoping Review

**DOI:** 10.1089/jmxr.2024.0012

**Published:** 2024-07-03

**Authors:** James S. Cho, Devaunsh M. Thaker, Rohan Jotwani, David Hao

**Affiliations:** 1 Department of Anesthesia, Critical Care and Pain Medicine, Massachusetts General Hospital, Boston, Massachusetts, USA.; 2 Harvard Medical School, Boston, Massachusetts, USA.; 3 Department of Anesthesiology, Perioperative Care, and Pain Medicine, NYU Langone Health, New York, New York, USA.; 4 Department of Anesthesiology, Weill Cornell Medicine, New York, New York, USA.

**Keywords:** spatial computing, navigation, augmented reality, virtual reality, mixed reality, extended reality, neuraxial, spinal, epidural, anesthesia, pain, simulation, education

## Abstract

**Background:**

Extended reality (XR) technology, encompassing augmented reality, mixed reality, and virtual reality, has the potential to enhance the teaching and performance of neuraxial procedures. The diverse applications of XR include immersive simulations and novel modes of procedural navigation.

**Objectives:**

This scoping review aims to explore the varied applications of XR for neuraxial procedures while suggesting directions for future research.

**Evidence Review:**

A systematic search was conducted across PubMed, Embase, Web of Science, Cochrane Central Register of Controlled Trials, and Google Scholar until December 2023. Additional sources were identified via citation searching of relevant articles. The findings are reported using the Preferred Reporting Items for Systematic Reviews and Meta-Analyses extension for Scoping Reviews (PRISMA-ScR).

**Findings:**

A total of 42 studies, including three pending clinical trials were included. Most included studies were published after 2015. XR technology was applied in diverse ways for simulation (*n* = 24), navigation (*n* = 17), and teleconsultation (*n* = 1), but only five of the completed studies described use in direct patient care. For the display of visuals, computer screens were most commonly used, followed by head-mounted devices, laser projectors, and semi-transparent mirrors.

**Conclusions:**

Interest in using XR technology for neuraxial procedures is growing. Preliminary work shows promise for this technology in both education and clinical practice, but achieving accurate image registration without disrupting existing workflows remains an ongoing barrier to clinical testing. Additional research is needed to assess the cost-effectiveness and reliability of this technology.

## Introduction

Extended reality (XR) holds significant potential in medical education and clinical practice.^1–3^ XR can be used to create immersive simulations or enhance standard modes of anatomical navigation.^2^ Despite these potential benefits, the use of XR within anesthesiology and pain medicine remains limited.^4^

### The XR spectrum

XR is an umbrella term that encompasses augmented reality (AR), mixed reality (MR), and virtual reality (VR).^5^ In AR, digital images are overlaid onto the physical world, annotating the world with contextual data.^6^ In MR, digital objects behave like physical objects, allowing a high degree of interactivity.^5^ VR, on the other hand, can be defined as a “three-dimensional computer-generated simulated environment, which attempts to replicate the real world or imaginary environments and interactions.”^7^

XR can be achieved in numerous ways. Some modern head-mounted devices (HMDs) offer visual passthrough, a feature that allows the wearer to view the real world through a digital screen via cameras and sensors.^8^ These devices can allow variable immersion to fit the needs of the application and the user, in effect moving wearers through the XR spectrum. Other devices achieve XR by presenting digital overlays on transparent displays.^9^ This maintains a real-time view of the surroundings without constraints imposed by cameras and nontransparent digital displays. XR can even be achieved without the use of screens, such as by using projectors,^10^ although this potentially limits the ability to view three-dimensional images.

### XR for neuraxial procedures

Neuraxial procedures are commonly performed to treat chronic pain^11^ or to provide anesthesia and analgesia during surgery^12^ and labor.^13^ These procedures involve accessing the epidural or intrathecal space and include epidural injections^11^ and spinal anesthesia.^14^ Today, neuraxial procedures are commonly taught and performed using landmark-based approaches or with the use of ultrasound or fluoroscopy.^11,15^ However, each method has limitations related to accuracy, ergonomics, or radiation exposure. The evolution of XR technology, combined with improved access to commercial XR devices, could pave the way for innovative methods of teaching and performing neuraxial procedures. With sufficient development and validation, XR technology may improve the quality of education and patient care. However, the current progress in adopting this technology for neuraxial procedures has not been systematically analyzed.

### Objectives and review questions

This scoping review aims to summarize the current applications of XR for neuraxial procedures and to identify barriers to widespread use. The following questions were defined to inform our search strategy:

1. How has XR been used for neuraxial procedures in clinical settings?
2. How has XR been used for training clinicians to perform neuraxial procedures?
3. What are the benefits of and barriers to adopting XR for teaching or performing neuraxial procedures?
4. What are clinician attitudes toward adopting XR for teaching or performing neuraxial procedures?

## Methods

A protocol for this scoping review was preregistered on the Open Science Framework on November 5, 2023.^16^ The reporting of this scoping review was guided by the standards of the Preferred Reporting Items for Systematic Reviews and Meta-Analyses extension for Scoping Reviews (PRISMA-ScR).^17^

### Search strategy and sources of evidence

A systematic search of the literature was performed on PubMed, Embase, Web of Science, Cochrane Central Register of Controlled Trials, and Google Scholar. The search query combined ([augmented OR extended OR mixed OR virtual] AND reality) AND (epidural OR peridural OR intrathecal OR spinal OR neuraxial) AND (anesthesia OR pain). No filters were applied. Owing to a high volume of irrelevant results on Google Scholar (greater than 200,000 results), we initially screened the first 100 results for relevance, and later expanded to include 20 additional results in the most recent search on December 14, 2023. Additional references were identified through citation searching of relevant articles. Pending trials identified via Cochrane Central Register of Controlled Trials were periodically monitored by following updates on the relevant clinical trial registry, and the most recent trial included was published on March 29, 2024. The search strategy used for each database is provided in the online Supplementary Appendix SA1.

### Eligibility criteria

The PICO (population, intervention, comparison, and outcomes) framework was used to develop the inclusion and exclusion criteria (Table 1). All primary studies available in English and investigating the use of XR in the performance or teaching of neuraxial procedures were included. Technical reports and conference papers were also considered. No restrictions were imposed on the publication year. Although review articles were ultimately excluded, each related review underwent thorough evaluation, and relevant citations from these reviews were incorporated into the screening pool. Essays and letters describing theoretical applications were excluded.

**Table 1.** Inclusion and Exclusion Criteria

	Inclusion criteria	Exclusion criteria
Population	Clinicians and trainees performing or learning neuraxial procedures; patients receiving neuraxial procedures	No additional limitations
Intervention	Use of extended reality to aid the teaching or performance of neuraxial anesthesia or neuraxial pain procedures	Use of extended reality by patient (e.g., for anxiolysis during a procedure)
Comparison	All comparisons	No limitations
Outcomes	All outcomes	No limitations
Study design	Any primary investigations	Reviews^a^ and essays or letters describing theoretical applications
Years	Any	No limitations
Language	English full-text available	English full-text not available

a Reviews were excluded, but relevant bibliography was screened and included as appropriate.

### Study selection

After importing all retrieved articles into Covidence (Melbourne, Australia), automatic and manual deduplication was performed. Two authors (J.S.C. and D.M.T.) screened the articles initially by title and abstract, followed by a full-text review. When access to full-text was unavailable, corresponding authors were contacted for access. A third author (D.H.) was available to provide resolution if discrepancies could not be resolved via discussion.

### Data extraction

For each article, two authors (J.S.C. and D.M.T.) manually extracted the following data into predesigned forms on Covidence: title, authors, year of publication, first author country, study design, purpose of XR system, mode of XR, XR display device used, procedure taught or performed, number of participants and description, key outcomes, clinician attitudes regarding adoption of XR, limitations of described system, and suggestions for further research. For this review, AR and MR were grouped into one category, as explained later in the study. Study designs were often not explicitly stated and were manually coded by the authors.

To maximize congruence, we used an iterative approach where the two extractors periodically compared their results (initially after each study and subsequently after every few studies), achieved consensus, and discussed strategies to maximize accuracy. As for screening, a third author (D.H.) was available to resolve disagreements.

### Synthesis of results

We grouped the included studies by the purpose for which XR applications were created and summarized the device(s) used to achieve XR, the neuraxial procedure(s) performed, the population studied, and key findings. Participants’ responses to the use of XR as well as notable limitations of the described system were also summarized. Study authors’ suggestions for further research were also tabulated.

## Findings

**FIG. 1.**
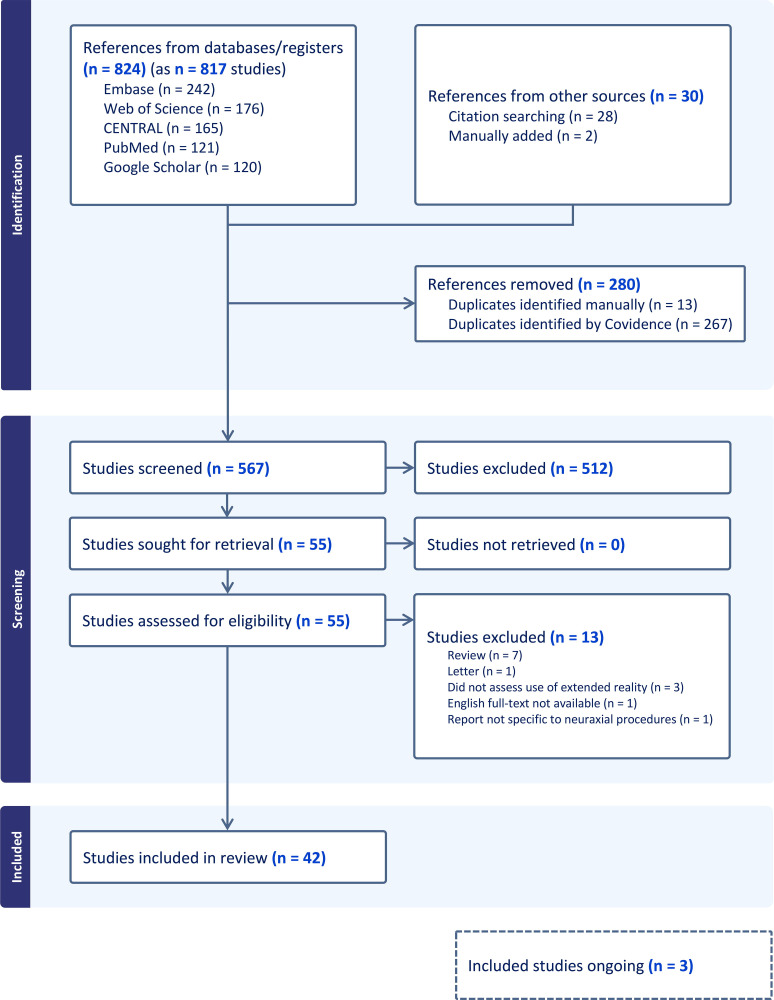
PRISMA flowchart of the review process.

A PRISMA flowchart illustrating our review is shown in Figure 1. Our initial search yielded 824 entries, and citation searching led to the addition of 28 additional references. One unindexed article^18^ cowritten by one of the authors (R.J.) was manually included, as it met inclusion criteria. Another article published after search completion was manually added.^19^ A total of 280 duplicates were removed. After screening of titles and abstracts, 55 articles were selected for full-text retrieval. All 55 full-text articles were successfully retrieved (three after contacting the authors), and 42 studies were ultimately included in this review. References pertaining to the same study were merged.

A total of 13 articles were excluded.^4,20–31^ Three were deemed irrelevant as XR was not used.^20–22^ One article was not available in English and was excluded.^23^ A letter describing the theoretical impact of XR on obstetric anesthesia was also excluded.^24^ Seven reviews were excluded,^4,25–30^ but relevant citations were incorporated into the screening pool. One article was excluded as the reported progress (i.e., viewing educational content using an HMD) was not considered specific to teaching neuraxial procedures.^31^

The characteristics of the included evidence are displayed in Table 2. We grouped our studies into three categories. The first category includes studies where XR was used to develop or enhance simulators (*n* = 24).^18,32–54^ The second category includes 14 completed studies^19,55–67^ where XR was intended to be used for procedural navigation, as well as three pending studies.^68–70^ The final category includes one study^71^ in which XR was used for teleconsultation. Use of XR in direct patient care was infrequent, with only five^18,19,54,61,71^ of 39 completed studies describing such use.

**Table 2.** Characteristics of Included Studies

Characteristic	Value	*N* (%)
First author country	Canada	11 (26.2)
	United States	10 (23.8)
	China	6 (14.3)
	South Korea	5 (11.9)
	United Kingdom	3 (7.1)
	Germany	2 (4.8)
	Brazil	1 (2.4)
	Ireland	1 (2.4)
	Japan	1 (2.4)
	Portugal	1 (2.4)
	Singapore	1 (2.4)
Year of publication	Before 2000	2 (4.8)
	2000–2010	1 (2.4)
	2011–2020	18 (42.9)
	2021–2024	21 (50.0)
Form of XR	AR/MR	23 (54.8)
	VR	16 (38.1)
	Both AR and VR	3 (7.1)
Intended Use of XR	Simulation	24 (57.1)
	Navigation	17 (40.5)
	Teleconsultation	1 (2.4)
Study design	RCTs	6 (14.3)
	Planned RCTs	3 (7.1)
	Non-RCT experiments	17 (40.5)
	Case report/series	4 (9.5)
	Technical report / usability	12 (28.6)
XR device	Computer screen	18 (42.9)
	Head-mounted device	17 (40.5)
	Semi-transparent mirror	2 (4.8)
	Projector	2 (4.8)
	Unspecified	3 (7.1)
Source of funding	Not reported	9 (21.4)
	Not sponsored	4 (9.5)
	Industry	4 (9.5)
	Public, department, or nonprofit	25 (59.5)

AR, augmented reality; MR, mixed reality; RCT, randomized controlled trial; VR, virtual reality; XR, extended reality.

Various study designs were identified, including randomized controlled trials (RCTs) (*n* = 6),^19,39,42,46,49,61^ planned RCTs (*n* = 3),^68–70^ non-RCT experiments (*n* = 17),^37,40,47,50,52,53,55–59,62–67^ case reports and series (*n* = 4),^18,41,54,71^ and technical reports (*n* = 12).^32–36,38,43–45,48,51,60^ Only four studies reported industry support (either funding or devices).^32,35,44,60^ Four studies were unsponsored,^45,46,52,54^ and 25 studies only reported public, departmental, or nonprofit funding.^19,38–40,42,43,47–51,53,55,56,58,59,61–69^ Nine studies did not report sources of funding.^18,33,34,36,37,41,57,70,71^

Researchers from Canada^40,42,47,48,55–61^ and the United States^18,32,35,37,41,51–53,62,63^ were first authors for 50% of the included studies, but those from Europe,^33,34,36,43,44,66,71^ China,^45,50,67–70^ and South Korea^19,49,54,64,65^ have made substantial contributions: 92.9% of studies were published (or registered, in the case of ongoing studies) after 2010,^18,19,33,36–67,71^ and 50% were published/registered after 2020.^18,19,43–46,48–52,54,60,61,64–67,71^ A total of 18 completed studies^32–42,48,51–53,55,56,59^ used computer screens to display XR visuals, and 17 used HMDs.^18,19,43–47,49,50,54,60,61,64,65,67,71^ The remaining four studies used either laser projectors^57,58^ or semi-transparent mirrors^62,63^ to project relevant visuals onto the subject’s back.

A summary of each study is provided in Supplementary Table S1 and discussed below.

### XR for developing or enhancing simulators

Of the included studies, XR was most commonly used to develop or enhance neuraxial simulators.

Before the release of commercial XR devices, applications of XR for neuraxial simulation involved nonimmersive VR, presenting a virtual patient^32–36^ or target^37^ on a computer screen for a simulated neuraxial procedure. Haptic devices controlled the virtual needle and provided feedback as the needle traversed simulated tissues of varying density. Attempts to enhance realism of the feedback incorporated force data derived from porcine models,^32,33^ CT data,^34^ and MRI intensity levels.^35^ In order to increase engagement, some iterations incorporated gamification, or the use of game design elements in nongaming contexts,^72^ by awarding points^34^ and virtual “achievements.”^38^

Kulcsár et al. conducted a pilot randomized-controlled trial using a VR spinal anesthesia simulator.^73^ Subjects viewed a virtual back through a 3D display while wearing shutter glasses and controlled a needle using a haptic device. The results of training on this system were compared with those undergoing low-fidelity training on an orange.^39^ Although no significant differences were observed in multiple-choice and simulator-based tests, simulator-trained interns exhibited better performance on real patients according to a global rating scale.^39^ This study was limited by its small sample size of 27 subjects, of which only 11 completed clinical testing.

One advantage of XR is its ability to provide contextual information that is typically unavailable, such as holographic visuals. In one study, an interactive spine model viewed on a computer screen was used to improve trainee knowledge through simulation of spinal ultrasound scanning without the need for a real ultrasound machine.^40^ Edwards et al. applied an MR simulator in which 3D images were visualized on a computer screen to analyze how the procedure needle interacts with internal anatomy.^41^ Using this simulator, the researchers developed a modified paramedian thoracic epidural technique, which was taught to trainees and then applied in challenging patients.^41^ The implications of this study are unclear, owing to its small sample size and lack of a control group.

In a 2015 RCT, Keri et al. evaluated an AR system on learners practicing ultrasound-guided lumbar puncture on a mannequin.^42^ The user could see 3D representations of ultrasound images and the needle overlaid onto a spine model, which was visualized on a computer screen. The study found that residents who practiced ultrasound-guided lumbar puncture with AR enhancement later outperformed those who practiced with ultrasound alone.^42^ As the authors report, it is unknown whether the learning benefits demonstrated in this study would translate to the clinical setting, as post-training testing also occurred on the mannequin.

Hologram-producing HMDs have found use in neuraxial education. Usability studies using HoloLens (Microsoft Corporations, Redmond, Washington) for training garnered positive responses from participants.^43–46^ However, an RCT conducted by Hayasaka et al. failed to demonstrate a lasting difference between students trained on an AR-enhanced phantom and those trained without AR.^46^ In this study, 30 medical students were divided into conventional, AR, and semi-AR groups, and their ability to perform an epidural procedure using a predefined ideal technique was measured. Although those using AR performed better while AR was enabled, a posttest without AR could not distinguish them from those who trained without AR.^46^

Most recent VR applications were built using the Unity game engine (Unity Software Inc., San Francisco, California).^38,47–50^ Unity simplifies the development of XR applications and reduces duplicate work via software plug-ins used to implement XR-related features for various devices.^74^ In one RCT involving a Unity-based immersive VR simulator, trainees learning lumbar transforaminal epidural block placement using an HMD outperformed those who learned via text and video material alone.^49^ As with many similar studies, participants could not be blinded to the intervention, and the clinical implications are unclear, given the use of a phantom for the posttest.^49^

Although software like Unity has reduced the redundant work in the development of XR education and simulation software, there is significant heterogeneity in how individual systems are developed. Lampotang et al. argue that these heterogeneous approaches may lead to increased validation and curriculum development costs. They describe the System of Modular, Mixed and Augmented Reality Tracking Simulators (SMMARTS) as a potential solution.^51^ SMMARTs enables the reuse of hardware and software components and allows for the development of varied procedural simulators, including an epidural loss-of-resistance simulator.^51^ The system offers a software development kit built with Unity to facilitate development and hardware-software integration.^51^

Our search strategy identified one validation study of commercial XR simulators. White and Jung conducted a pre- and posttest study on 14 trainees using a spinal cord stimulator training system.^52^ After 7 min of instruction using the VR simulator, which combined a computer display for simulating fluoroscopy images as well as a model of a sample patient’s surface anatomy, participants had subjective and objective improvements in their performance.^52^ A notable limitation was the $50,000 cost of the system (SPINE Mentor, Surgical Science, Göteborg, Sweden).^52^ Although this system appears to offer a high fidelity simulation of spinal cord stimulator lead placement, the small sample size and the lack of a control group in this study limit an assessment of its cost-effectiveness.

An atypical use case for XR neuraxial simulators was presented by Cometa et al.^53^ They compared the epidural space overshoot (the additional distance traveled by the epidural needle tip after achieving loss-of-resistance) associated with different loss-of-resistance techniques using measurements derived from an MR-based simulator. Intermittent needle advancement with intermittent plunger pressure resulted in the most significant overshoot.^53^ As demonstrated by this study, simulators can construct experimental conditions difficult to mimic *in vivo*, but the clinical transferability of simulator-derived results is variable.

#### Preprocedure simulation

VR has successfully been used for preprocedure planning in real patients undergoing challenging interventional procedures. A case report by Wang et al. describes the successful use of an existing VR anatomy application, accessed using an HMD, to determine the optimal fluoroscopic angulation in a patient with a suspected schwannoma undergoing a transforaminal epidural injection at the level of the lesion.^18^ Seong et al. developed VR software enabling clinicians to plan and simulate needle interventions using patient computed tomography (CT) scans.^54^ This system allowed clinicians to simulate fluoroscopy and obtain x-ray images representative of the real patient to determine the ideal approach. Clinicians successfully executed a challenging transforaminal epidural injection following prior failed attempts by implementing the preprocedure plan designed in VR.^54^

### XR for procedural navigation

Fourteen studies^19,55–67^ have investigated whether XR can be used to enhance navigation during neuraxial procedures. The described systems differed in whether XR images were generated based on ultrasound, cross-sectional imaging, or fluoroscopy. Only two^19,61^ of the studies evaluated XR navigation on patients, but three additional clinical trials are planned.

#### Ultrasound

A significant focus was placed on improving ultrasound-assisted neuraxial procedures using AR and MR. Three studies used image processing algorithms to automatically detect and project lumbar spine levels^55,56^ or the midline^57^ during neuraxial ultrasound, either on a computer screen^55,56^ or onto the subject’s body^57^ using a projector. A fourth study introduced a machine learning/artificial intelligence-based system for automatic detection and laser projection of spine levels.^58^ The authors suggest such systems could be used clinically by anesthesiologists to determine the appropriate site of initial needle insertion. However, whether these systems would confer benefit for anesthesiologists with training in spinal ultrasound is unclear.

Continuous ultrasound guidance during needle placement can present ergonomic and visual challenges. Ameri et al. reported a possible solution, using a tracked epidural needle and AR to improve visualization of the needle and sonoanatomy on ultrasound.^59^ This system showed a lower rate of accidental dural punctures compared with using only ultrasound in trials conducted on a phantom.^59^

Tanwani et al. also described the use of a tracked ultrasound, this time to determine and project the optimal needle trajectory from the skin to the epidural space. This trajectory was visualized as a hologram by the proceduralist using an HMD.^60^ In an RCT including 83 patients, Wiegelmann et al.^61^ assessed whether this system can reduce time to thoracic epidural catheter insertion in patients presenting for thoracic or abdominal surgery. This study found a significant reduction in average procedure time with XR compared with traditional approaches (4.5 min vs. 7.3 min, *p* = 0.02).^61^ Although there was a significant reduction in the number of needle movements required (7.2 vs. 14.4, *p* = 0.01), there was no noticeable difference in the pain associated with the procedure. The authors recommend additional study to determine if the benefits measured lead to clinical benefits, such as reduced epidural failure rates.

#### CT/MRI

One VR planning and AR navigation system was developed and tested on torso phantoms^64,65^ before undergoing a feasibility study in an RCT.^19^ This system used preprocedural CT scanning to enable VR navigational planning on a computer screen and AR-assisted navigation using an HMD during transforaminal epidural injection. In Jang et al.,^19^ 28 patients were randomized into a control group with C-arm guidance and an AR navigation guided group (AR-NAVI). Patients in the AR-NAVI group experienced significantly shorter procedural times (mean 86.86 s vs. 195.2) and C-arm uses (5.07 vs. 13.29).^19^ Although this study is limited by lack of proceduralist blinding and the evaluation of only one proceduralist, it demonstrates the potential for AR navigation systems to increase the efficiency and safety of certain neuraxial procedures. The authors call for a larger array of randomized controlled trials and multicenter studies.^19^

Fritz et al. addressed limitations of magnetic resonance imaging-guided lumbar spine interventions by overlaying preprocedural axial magnetic resonance images onto the procedural field using a semi-transparent mirror.^62,63^ With this system, the proceduralist could view cross-sectional imaging and planned needle trajectories at the correct location on the target. This system enabled successful epidural injections on cadavers without repeat scanning, with a median procedure time of 8.6 min.^63^ Although the system was tested on phantoms and cadavers, these studies have potential clinical implications as the described techniques seem feasible on patients, provided that patient respiratory movements do not significantly affect the accuracy of registration.

Reinacher et al. demonstrated high success rates with HMD-based AR in simulated thoracolumbar neuraxial access on prone phantoms.^66^ In this study, first pass epidural entry rate was 40% with a landmark approach versus 82.5% for the AR approach. The mean Euclidean distance to the planned target was 6.1 mm in the AR group, compared with 12 mm in the landmark group. This study has several limitations. The phantom did not simulate *in vivo* characteristics used to identify the epidural space, such as the variable resistance of tissues experienced by the proceduralist.^66^ The compared outcomes may not have clinical relevance, as neither a greater first pass success rate nor smaller distance to an *ideal* target guarantee superior results.

Wu et al. described the use of a posture fixation device to address the challenges of achieving accurate image generation and maintaining reliable registration.^67^ In a study with volunteers, researchers assessed the accuracy of overlaying holograms of spinous processes on their actual locations.^67^ CT images were used to generate three-dimensional images of the spine, which were projected over the subject’s backs using an HMD.^67^ Markers were placed over the holographic spinous processes, and the subjects underwent a repeat CT scan to assess the accuracy of marker placement. The maximum mean error along one axis was 4.2 mm.^67^ A significant limitation of this system is that, for clinical use, patients would need to undergo preprocedural CT scanning while using a posture fixation device and maintain that position during the neuraxial procedure, but this limitation also contributes to the system’s accuracy.

#### Pending studies

Our review includes three uncompleted clinical trials, all registered by the same group of investigators and listed as “not yet recruiting” according to the Chinese Clinical Trial Registry.^68–70^ The researchers aim to compare the success rates of first-pass lumbar puncture for clinicians using MR guidance versus those using the conventional approach. Efforts to contact the researchers for updates on the studies were unsuccessful. The group’s prior work^67^ was discussed earlier in this section.

### XR for teleconsultation

This review identified one instance of AR-enabled remote consultation during spinal cord stimulator surgery. Fritz et al. detail the use of AR goggles which enabled a remote specialist, located 200 miles away, to observe the procedure and provide annotated images and live commentary.^71^ This led to the successful insertion of paddle leads in a patient with highly challenging anatomy.^71^

## Discussion

**FIG. 2.**
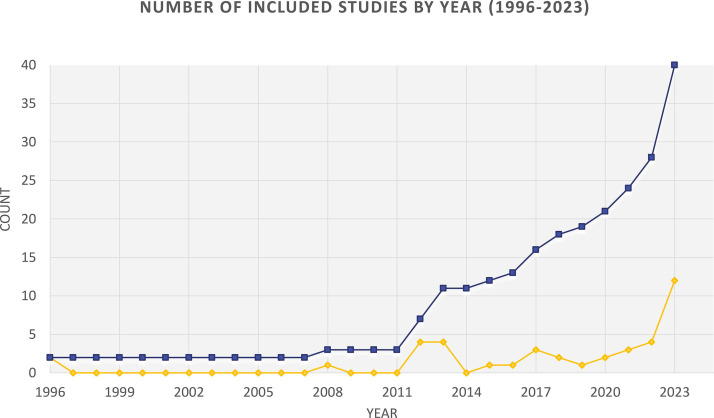
Number of included studies by year (1996 to 2023). Blue line: Total number of studies over time. Yellow line: Studies added per year.

To our knowledge, this is the first scoping review on the uses of XR technology for neuraxial anesthesia and neuraxial pain procedures. We identified 39 completed and three pending studies investigating the use of XR for neuraxial procedures. Our findings demonstrate accelerating interest in the use of XR technology. The first included article was published in 1996, but a significant majority was published after 2015, as shown in Figure 2. As demonstrated in Supplementary Table S1, a growing proportion of studies used commercial XR devices as the primary display device. Certain devices, such as the HoloLens were widely applied, likely due to their technical capabilities and robust software development pipelines. The release and widespread adoption of newer commercial, standalone devices such as the Meta Quest 3 (Meta Inc., Menlo Park, CA) and the Vision Pro (Apple Inc., Cupertino, CA) is expected to further drive XR research.

Although a significant number of studies have been conducted in Canada and the United States, our results indicate global interest in XR for neuraxial procedures. However, when accounting for duplicate authors, the total number of active research groups in this field remains limited.

To avoid confusion stemming from misuse of terminology, we intentionally grouped AR and MR in our analysis. AR, MR, and VR exist on a spectrum,^75^ and modern devices often support multiple forms of XR. This makes functional categorization (e.g., simulation vs. procedural navigation) more relevant than the relative visibility of virtual and physical content.

A majority of the included studies evaluated the use of XR in educational settings. Surveyed trainees and clinicians displayed favorable responses to the adoption of XR for neuraxial education.^30,35,37,43,44,46^ The largest reported survey of trainees and anesthesiologists included more than 90 respondents, and VR simulation was highly rated on various metrics, including ease of use (median score eight out of ten) and visual quality (median score eight out of ten).^35^ However, there is limited research investigating whether skills developed via XR-based training transfer to clinical care.

Patient-facing use of XR for neuraxial procedures remains limited. This is in contrast to specialties such as neurosurgery, where AR is used clinically for intraoperative navigation.^76^ Similar application of XR navigation would confer numerous benefits in neuraxial procedures. If patient-specific anatomical holograms can be precisely overlayed onto the procedural field, XR may allow intuitive and hands-free navigation (vs. ultrasound), improve accuracy (vs. blind procedures), and reduce radiation exposure (vs. fluoroscopy). Indeed, initial clinical RCTs included in this review^19,61^ each noted a benefit or combination of benefits listed above.

### Barriers to widespread adoption of XR

Despite these potential benefits, certain barriers hinder the widespread adoption of XR technology for neuraxial navigation. The cost of XR systems can vary widely depending on system complexity, and how clinicians and patients will respond to nontraditional navigation modes is still unknown. However, the most significant barrier lies in displaying accurate navigational information for each individual patient. Preprocedural cross-sectional imaging is often unavailable for patients undergoing neuraxial procedures. Even when imaging is available, patient positioning can dramatically affect the shape of the spine, making image-to-patient registration challenging. Existing research suggests that it is possible to incorporate XR into ultrasound-assisted neuraxial procedures with minimal impact on workflow.^61^ However, clinical validation for fluoroscopic-guided procedures without the use of an alternative modality for XR image generation has not been performed.

### Future directions

This review has identified several gaps in the literature. There should be greater focus on developing XR systems for use in direct clinical care as opposed to use in simulation. Despite several years of active research, there is paucity of data supporting the use of XR in patient-facing settings, as most of the proof-of-concept techniques described have not undergone clinical validation. These systems should be refined to accommodate constraints faced in clinical care, such as those related to maintaining sterility or the availability of imaging and hardware. Greater clinician involvement during the conceptualization and evaluation processes would aid in promoting compatibility with clinical workflows. Each system should also undergo rigorous evaluation via randomized controlled trials, as modeled by Jang et al.^19^ in their feasibility study in real patients using techniques described by Lim et al.^64^ and Jun et al.^65^

Currently, there is insufficient evidence to recommend routine imaging for landmark-based procedures solely for the purpose of allowing XR navigation. As such, procedures most amenable to XR-enhanced navigation are those for which intraprocedural imaging is readily available, such as ultrasound-guided neuraxial procedures. Efforts to retain the benefits of imaging guidance while minimizing the downsides are most likely to facilitate clinical adoption, particularly when applied to patients with atypical anatomy.

As demonstrated by Hetherington et al.’s application of machine learning,^58^ there is potential for artificial intelligence to enable new ways of developing XR systems. With the use of artificial intelligence, clinicians will be able to apply existing data in novel ways through computer interpretation and processing. The authors envision a major role for artificial intelligence in the generation of XR images and also in allowing improved dynamic registration of XR images during procedures.

### Limitations

This scoping review has several limitations. Although our search and screening criteria allowed for the inclusion of diverse studies published in various forms, there is likely to be publication bias in the included studies, many of which report favorable results. A formal assessment of the quality of the evidence is beyond the scope of a scoping review, and, as such, we do not provide practice recommendations.

Our study deviated slightly from the preregistered protocol in a few instances. During the screening process, we decided to exclude studies not available in English, leading to the exclusion of one study. In addition, we excluded reviews, although relevant citations were included. Originally, we did not anticipate the need to contact the authors of individual studies, but we ultimately contacted three authors to obtain full-text articles. We believe that these deviations had minimal or even positive impacts on the review.

Despite these limitations, this scoping review offers a comprehensive overview of the available research on the use of XR for neuraxial procedures. Our broad and iterative search strategy led to the screening of a large number of studies, and our inclusive eligibility criteria led to the inclusion of diverse studies spanning nearly three decades. As intended, this scoping review maps the existing evidence, identifies knowledge gaps, and provides suggestions for further research.

## Conclusions

This scoping review emphasizes the diverse applications of XR technology in the teaching and performance of neuraxial procedures within the fields of anesthesiology and pain medicine. Although many of the included studies were exploratory in nature, there is a growing cohort of researchers investigating the potential of XR in clinical settings. To advance the field, future research should prioritize investigations into the safety, accuracy, practicality, and cost-effectiveness of XR. A concerted effort in these areas is expected to catalyze adoption of this technology and enhance the quality of both education and patient care.

## Author Contributions

J.S.C. and D.M.T. were responsible for the conception and design of the study, data acquisition, analysis, interpretation, and manuscript preparation. R.J. and D.H. provided critical feedback on the study design and contributed to data analysis, interpretation, and manuscript preparation.

## Supplementary Material

Supplementary Appendix SA1

## Abbreviations Used

2D: Two-Dimensional
3D: Three-Dimensional
AR: Augmented Reality
CAAs: Certified Anesthesiologist Assistants
CSE: Combined Spinal-Epidural
CRNAs: Certified Registered Nurse Anesthetists
CT: Computed Tomography
DOF: Degree-of-Freedom
LOR: Loss of Resistance
MRI: Magnetic Resonance Imaging
MR: Mixed Reality
N/A: Not Applicable
RCT: Randomized Controlled Trial
RMS: Root Mean Square
SMMARTS: System of Modular Mixed and Augmented Reality Tracking Simulators
US: Ultrasound
VR: Virtual Reality
XR: Extended Reality
